# Laboratory prediction of the requirement for renal replacement in acute falciparum malaria

**DOI:** 10.1186/1475-2875-10-217

**Published:** 2011-08-03

**Authors:** Josh Hanson, Md Mahtab Uddin Hasan, Annick A Royakkers, Shamsul Alam, Prakaykaew Charunwatthana, Richard J Maude, Sam T Douthwaite, Emran Bin Yunus, Murty L Mantha, Marcus J Schultz, M Abul Faiz, Nicholas J White, Nicholas P Day, Arjen M Dondorp

**Affiliations:** 1 Cairns Base Hospital, Cairns, Australia; 2 Mahidol-Oxford-Research Unit, Bangkok, Thailand; 3 Chittagong Medical College Hospital, Chittagong, Bangladesh; 4 Laboratory of Experimental Intensive Care and Anesthesiology, Academic Medical Center at the University of Amsterdam, The Netherlands; 5 Nuffield Department of Clinical Medicine, University of Oxford, UK; 6 Sir Salimullah Medical College, Dhaka, Bangladesh

## Abstract

**Background:**

Acute renal failure is a common complication of severe malaria in adults, and without renal replacement therapy (RRT), it carries a poor prognosis. Even when RRT is available, delaying its initiation may increase mortality. Earlier identification of patients who will need RRT may improve outcomes.

**Method:**

Prospectively collected data from two intervention studies in adults with severe malaria were analysed focusing on laboratory features on presentation and their association with a later requirement for RRT. In particular, laboratory indices of acute tubular necrosis (ATN) and acute kidney injury (AKI) that are used in other settings were examined.

**Results:**

Data from 163 patients were available for analysis. Whether or not the patients *should *have received RRT (a retrospective assessment determined by three independent reviewers) was used as the reference. Forty-three (26.4%) patients met criteria for dialysis, but only 19 (44.2%) were able to receive this intervention due to the limited availability of RRT. Patients with impaired renal function on admission (creatinine clearance < 60 ml/min) (n = 84) had their laboratory indices of ATN/AKI analysed. The plasma creatinine level had the greatest area under the ROC curve (AUC): 0.83 (95% confidence interval 0.74-0.92), significantly better than the AUCs for, urinary sodium level, the urea to creatinine ratio (UCR), the fractional excretion of urea (FeUN) and the urinary neutrophil gelatinase-associated lipocalcin (NGAL) level. The AUC for plasma creatinine was also greater than the AUC for blood urea nitrogen level, the fractional excretion of sodium (FeNa), the renal failure index (RFI), the urinary osmolality, the urine to plasma creatinine ratio (UPCR) and the creatinine clearance, although the difference for these variables did not reach statistical significance.

**Conclusions:**

In adult patients with severe malaria and impaired renal function on admission, none of the evaluated laboratory indices was superior to the plasma creatinine level when used to predict a later requirement for renal replacement therapy.

## Background

Acute renal failure (ARF) is a relatively common complication of falciparum malaria in adults. In patients with severe disease the incidence of ARF may be as high as 45% [1,2] and is associated with a mortality rate of up to 75% [3].

The specialist care of patients with malaria-associated ARF (MARF) in Vietnam led to a marked decline in mortality from 75% to 26% [3]. The most important component of this intervention was the introduction of peritoneal dialysis, which was effective in reducing mortality even when given for a relatively short duration. Subsequently, it was shown that haemofiltration was superior to peritoneal dialysis in MARF and was also cost-effective, despite the greater initial outlay costs [4]. In the initial Vietnam series, 21% of patients receiving peritoneal dialysis for MARF died, the majority within 24 hours of starting peritoneal dialysis - the authors suggested that the earlier initiation of renal replacement therapy (RRT), may have prevented some of these deaths [3].

The pathology of MARF is that of an acute tubular necrosis (ATN) associated with localization of host monocytes in the kidney as well as sequestration of parasitized red blood cells in the microcirculation [5], although the precise mechanism of renal injury is not known. Patients who have ATN will usually require early RRT, however on admission to the hospital it is difficult to distinguish patients with ATN from patients with reversible pre-renal causes that will respond, albeit at different rates, to simple rehydration and effective anti-malarial treatment [6].

At present, the decision to initiate RRT is usually based on the clinical assessment of treating physicians. It is generally guided by a patient's response to therapy during the early stages of their admission, and is often influenced by the limited availability of trained staff, available facilities and economic factors. Therefore, the initiation of RRT is often delayed until the patients have failed to respond to rehydration and anti-malarial chemotherapy. This may be too late [3].

Even in resource-rich settings the differentiation of reversible pre-renal ARF from established ATN is challenging at initial presentation. This is because of a lack of uniform diagnostic criteria for ATN. Definitive morphological diagnosis with biopsy is rarely justified and improvement in renal function following fluid repletion is a retrospective inference. Therefore, several laboratory tests have been devised that might help differentiate these populations. They are based on assessment of the renal response to reduced perfusion, which is the avid retention of salt and water in an attempt to preserve the circulating volume. Proposed tests include: the renal failure index (RFI), the fractional excretion of sodium (FeNa), the fractional excretion of urea (FeUN), the urea to creatinine ratio (UCR), the urine to plasma creatinine ratio (UPCR), and the urinary sodium (UNa) and osmolality (UOsm) [7-11] (Table 1). However such tests are not uniformly accepted [12] and they have not been assessed systematically in the prediction of the requirement for RRT [13].

**Table 1:** Laboratory tests used to differentiate pre-renal ARF from ATN [7-11]

Test	Formula used to calculate
Renal failure index (RFI)	

Fractional excretion of sodium (FeNa)	

Fractional excretion of urea (FeUN)	

Plasma urea to creatinine ratio (UCR)	plasma urea/plasma creatinine

Urinary to plasma creatinine ratio (UPCR)	urinary creatinine/plasma creatinine

Urinary sodium concentration (UNa)	n/a

Urinary osmolality (UOsm)	n/a

RFI: > 1, evidence for ATN, FeNa: <1% evidence for pre-renal disease, >2% evidence for ATN, FeUN: <35% evidence for pre-renal disease, >50% evidence for ATN, UCR: >20 evidence for pre-renal disease,<15 evidence for ATN (n.b. non SI units), UPCR: >40 evidence of pre-renal disease, <20 evidence for ATN, UNa: <20 mmol/L evidence for pre-renal disease, >40 mmol/L evidence for ATN, UOsm: >500 mosm/kg evidence for pre-renal disease, <450 mosm/kg evidence for ATN. n/a: not applicable.

More recently several biomarkers of acute kidney injury have been described. The most promising of these is neutrophil gelatinase-associated lipocalcin (NGAL), which is secreted into the urine from the ascending loop of Henle and collecting ducts of the kidney in proportion to the degree of acute injury [14]. Urinary NGAL on admission is correlated with mortality and a later requirement for RRT in critically ill patients [15].

The aim of this study was to assess the ability of these tests of ATN/AKI to predict the later requirement for RRT in patients with severe falciparum malaria. Diagnostic tests that facilitate initiation of RRT might potentially improve outcome.

## Methods

The study was conducted at Chittagong Medical College Hospital, Bangladesh, a large tertiary referral hospital in the country's second largest city. The hospital serves a wide area in which malaria is endemic with peaks in transmission during the wet season. Transmission intensity is low and severe malaria is predominantly seen in adults.

Patients enrolled in clinical trials of adjuvant therapy of severe malaria between 2003 and 2007 were included in this study. From 2003 to 2005 patients were enrolled in a trial assessing N-acetylcysteine (NAC) [16]. In 2006 and 2007 the patients were drawn from those assessed for a trial examining the efficacy of levamisole (study ongoing). Informed consent was obtained from the families of all patients enrolled in the trials. All studies had been approved by the ethics committee of the Ministry of Health, Bangladesh and OXTREC, the ethics committee for studies in tropical countries of Oxford University, UK.

All patients had to satisfy pre-specified criteria for severe malaria (Table 2) and have asexual forms of *P. falciparum *on a blood film.

**Table 2:** Criteria for severe malaria (after Hien et al)[1]

**On admission**:	
1.	Cerebral malaria. GCS < 11.
2.	Shock. Systolic blood pressure <80 mmHg, with cool extremities
3.	Severe anaemia. Haematocrit <20%, with parasite count > 100,000/μl
4.	Liver disease. Total bilirubin > 42 μmol/L with parasite count > 100,000/μl
5.	Hyperparasitaemia. Peripheral parasite count >10%
6.	Acidosis. Venous bicarbonate <15 mmol/L
On admission, or developing within the first 72 hours:	
7.	Hypoglycaemia. Blood glucose <2.2 mmol/L
8.	Convulsions
9.	Pulmonary oedema
10.	Renal failure. Plasma creatinine > 265 μmol/L

Blood and urine were collected on study enrolment. These values were used to determine the laboratory indices. Malarial thick and thin films were read immediately and basic laboratory results (Plasma sodium, plasma potassium, blood urea nitrogen, blood glucose, plasma bicarbonate, plasma pH, haemoglobin, haematocrit) were available within minutes through the use of a portable hand held analyser (iStat with EC8+ cartridges, Abbott, Illinois, USA). Plasma and urine specimens were frozen at -80°C for subsequent biochemical analysis in Bangkok, Thailand. The urine NGAL was measured using a commercial ELISA (R&D Systems, Abingdon, UK) in Amsterdam.

The patients were managed according to the published WHO guidelines [17,18] and were resuscitated with normal saline, titrated against the clinical assessment of dehydration. Apart from the simple investigations available on admission (above), which were repeated when clinically indicated, none of the measures of ATN/AKI were available to the treating clinicians. There were two haemodialysis machines in the hospital; if these were unavailable peritoneal dialysis was provided where supervising staff and facilities were available.

A premorbid plasma creatinine was not available in any of the patients due to their remote location in a resource-poor setting. The usual classification of a fifty percent increase in the plasma creatinine could thus not be used to define acute renal failure. Accordingly acute kidney injury was defined as a creatinine clearance less than 60 milliltres per minute, a figure commonly used to define clinically significant renal dysfunction [19,20]. Creatinine clearance was estimated using the Cockcroft and Gault formula [21]. The case records were retrospectively reviewed by two independent internal medicine physicians (JH and PC) to determine whether patients should have received treatment with dialysis according to pre-defined criteria (Table 3). If there was disagreement, the chart was reviewed by an intensive care physician (AMD) and a consensus was reached

**Table 3:** Criteria used by reviewers to determine the requirement for dialysis (one or more required)

1.	Evolving hyperkalaemia (K greater than 5.5 mmol/L) and renal impairment.*
2.	Evolving acidosis (pH < 7.35, HCO3 < 15) and renal impairment.*

3.	Fluid overload not manageable by conservative measures and evolving renal impairment. *

4.	Uncontrolled seizures in the setting of renal impairment*

5.	Pericarditis in the setting of renal impairment*

6.	Dialysis initiated appropriately by treating physicians before above criteria were met (eg. significantly worsening renal impairment despite conservative measures)

* Renal impairment: BUN greater than 30 mmol/L or plasma creatinine > 200 μmol/L.

The results from the de-identified data were recorded in a computerised database (Microsoft Access, Microsoft Corporation, USA) and then analysed using statistical software (Stata 9.0, Statacorp, Texas, USA). The performance of the tests was assessed using receiver operating characteristic (ROC) curves. Dichotomous variables were analysed using the Chi squared, Fisher's exact and Kruskal-Wallis tests.

## Results

### Patients

**Figure 1:**
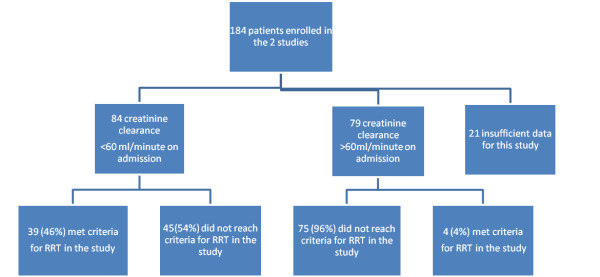
**Overview of the patients**.

Of the 184 patients assessed for these studies, 163 had sufficient data for analysis. Eighty four (51.5%) patients had a calculated creatinine clearance < 60 ml/min at study enrolment (Figure 1). Baseline characteristics of the patients are shown in Table 4.

**Table 4:** Baseline patient characteristics classified by admission creatinine clearance

Variable	All patients n = 163	CrCl > 60 ml/min n = 79	CrCl < 60 ml/min n = 84	P
Age (years)	35 (23 - 45)	30 (23 - 40)	37 (23 - 50)	0.02

Male sex	136/163 (80%)	66/79 (84%)	64/84 (76%)	0.33^

Parasite count % (peripheral)	2.7 (0.9 - 9.5)	1.9 (0.7 -4.9)	3.6 (1.1 - 11.3)	0.003

Glasgow Coma Score	8 (7 - 9)	8 (6 - 10)	9 (5 - 11)	0.8

Mean arterial pressure (mmHg)	80 (73 - 90)	81 (73 - 93)	80 (70 - 87)	0.2

Heart rate (beats per minute)	113 (102 - 131)	112 (94 - 131)	114 (103 - 134)	0.54

Respiratory rate (breaths per minute)	30 (26 - 36)	30 (25 - 36)	30 (26 - 36)	0.53

Oxygen saturation (%)	96 (94- 97)	96 (94 - 98)	96 (93 - 97)	0.61

Glucose (mmol/L)	6.8 (5.3 - 10.1)	7.2 (5.7 - 7.2)	6.3 (4.9 - 9)	0.09

Sodium (mmol/L)	133 (128 - 137)	132 (128 - 137)	133(129 - 139)	0.28

Potassium (mmol/L)	4.3 (3.8 - 4.8)	4.2 (3.7 - 4.6)	4.5 (3.8 - 4.9)	0.03

Bicarbonate (mmol/L)	16.9 (15 - 17.5)	19.3 (16 - 21.3)	14 (12 - 17.1)	0.0001

Base excess	-8 (-12 - -3)	-5 (-8 - -2)	-11 (-16 - -7)	0.0001

Lactate	4.7 (3.3-7.2)	4.3 (3.1 -5.5)	5.6 (3.7 -10.1)	0.002

Blood urea nitrogen (mmol/L)	14.7 (9 -26)	9.2 (6.4 - 12.1)	24.9 (18 - 34.3)	0.0001

Plasma creatinine (μmol/L)	122 (79.6 - 202)	79.2 (61.6 - 97.2)	202 (142 - 316)	0.0001

Haematocrit (%)	29 (23 - 35)	32 (26 - 36)	29 (23 - 35)	0.06

Urinary sodium (mmol/L)	44 (30 - 66)	48 (30 - 79)	40 (30 - 63)	0.35

FeNa	0.71 (0.28 - 1.37)	0.39 (0.2 - 0.84)	1.05 (0.52 - 2.11)	0.0001

RFI	0.95 (0.4 - 1.8)	0.5 (0.2 - 1.1)	1.3 (0.7 - 2.8)	0.0001

UOsm (mosm/L)	498 (391 - 674)	614 (503 - 725)	412 (357 - 518)	0.0001

FeUN	46 (29 - 65)	51 (38 - 66)	37 (24 - 64.5)	0.001

UPCR	53 (29.9 - 85.1)	80.5(60.3 - 115.8)	32.6 (19 - 46.9)	0.0001

Urea:creatine ratio	29.4 (21.8 - 37.8)	28.7 (20 - 38.4)	29.4 (22.4 - 37)	0.86

Plasma bilirubin (total) (μmol/L)	72 (35.9 - 160)	61.1 (29.9 - 111.3)	94.7(43.3 - 204.9)	0.01

Plasma bilirubin (direct) (μmol/L)	28.6 (12 - 77)	18.6 (10.4 - 42.8)	44 (18.8 - 113.3)	0.0001

NGAL	322 (226 - 586)	232 (171 - 333)	468 (304 - 733)	0.0001

All numbers, except male sex, are medians (interquartile range)

All P values comparing the populations with CrCl > 60 ml/min and CrCl < 60 ml/min are determined using Kruskal-Wallis, except ^ Fisher's exact.

CrCl: creatinine clearance, FeNa: Fractional Excretion of sodium, RFI: Renal Failure Index, UOsm: Urinary osmolality. FeUN: Fractional excretion of urea nitrogen, UPCR: urine:plasma creatinine ratio, UCR: plasma urea:creatinine ratio (n.b. non SI units), NGAL: neutrophil gelatinase-associated lipocalcin.

The patients were very unwell: 90% had a CAM score ≥2 [22] and sixty six patients (40.5%) died. Renal impairment was more common in older patients, those with a higher peripheral parasitaemia, a higher CAM score and an elevated plasma bilirubin. Predictably these patients were more likely to have a lactic acidosis and elevated plasma potassium.

**Figure 2:**
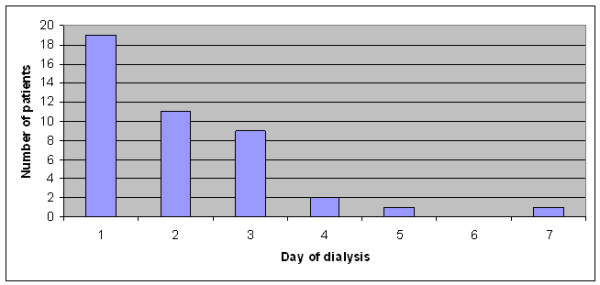
**Day of hospital admission that dialysis was commenced**.

There was 87% agreement between the internal medicine physicians in defining the patients with an indication for RRT. After discussion with the intensive care physician, there was unanimous consensus on the forty-three patients (26.4%) who should have received RRT. Twenty-four of these patients were unable to receive it for logistic reasons. Of the 19 patients who received RRT, seven patients (37%) died - a figure similar to the mortality rate in the series of patients without renal impairment (34%). However, in the 24 patients meeting criteria for RRT but who were unable to receive it, there were eighteen deaths (75%) - a statistically significant difference (p = 0.01). Two of the patients who received RRT had haemodialysis, the others received peritoneal dialysis. The majority of patients received RRT in the first 48 hours of admission (Figure 2). The duration of required RRT was relatively brief: surviving patients had dialysis for a median of three days (range 2-6 days). The most frequent indication for RRT was metabolic acidosis (Table 5)

**Table 5:** Indication for renal replacement therapy

Indication	Number
Renal impairment and acidosis	28

Renal impairment, acidosis and hyperkalaemia	8

Worsening renal impairment despite conservative measures	4

Worsening renal impairment and convulsions	1

Renal impairment and hyperkalaemia	2

Pericarditis	0

Convulsions	0

Total	43

**Figure 3:**
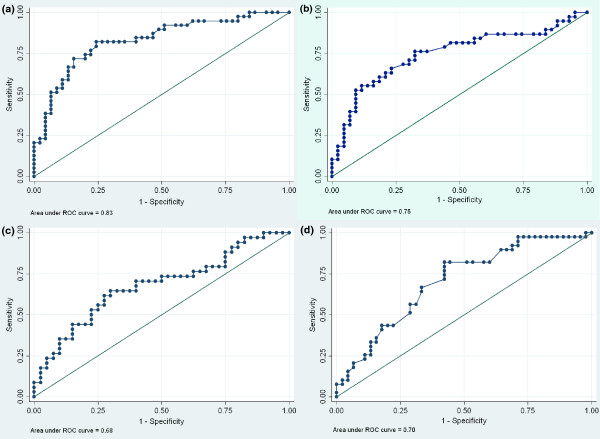
**Receiver operating characteristic curve showing the ability of variables to predict a later requirement for dialysis in those patients who should have received dialysis**. 3a) Plasma creatinine 3b) Fractional excretion of sodium 3c) Urinary neutrophil gelatinase-associated lipocalcin 3d) Plasma bicarbonate.

**Figure 4:**
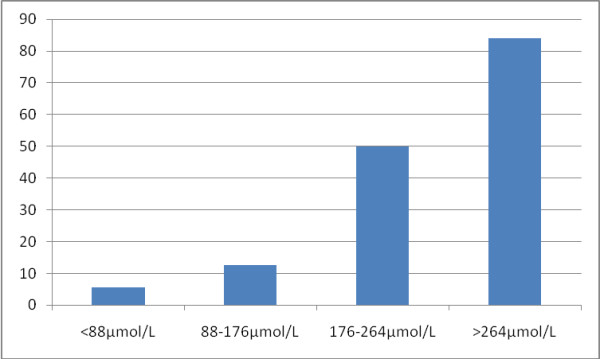
**Proportion who should have received RRT by admission serum creatinine**.

The data from patients with a creatinine clearance < 60 ml/min on admission (n = 84) were analysed to determine whether laboratory indices for ATN/AKI could predict a later requirement for RRT (Tables 6 and 7, and Figures 3a, 3b, 3c, 3d and 4). Measurement of the plasma creatinine on admission had the greatest ability to define a later requirement for RRT as determined by the area under the receiving operator characteristic curve (AUROC): 0.83 (95% confidence interval (CI) 0.74-0.92). If only the patients who actually received RRT were analysed admission plasma creatinine had a greater AUROC (0.69 (95% CI 0.55-0.83) than all laboratory indices except urinary osmolality (0.74 (95%CI 0.6-0.87), p = 0.37 for a difference).

**Table 6:** Receiver Operating Characteristic (ROC) analysis of the ability of various laboratory tests performed on admission to predict the later requirement for RRT in patients with an admission creatinine clearance of less than 60 ml/minute

Index	Median values in patients who should have received dialysis	Patients who were able to have dialysis		Patients who should have had dialysis		p*
				
		AUC	95% CI	AUC	95% CI	
Plasma creatinine (μmol/L)	290(226 - 379)	0.69	(0.55-0.83)	0.83	(0.74 - 0.92)	

Creatinine clearance (ml/min)	24.7 (20.7 - 31)	0.66	(0.51-0.81)	0.78	(0.68 - 0.88)	0.1

RFI	2.5 (1.5 - 3.6)	0.64	(0.48-0.8)	0.75	(0.64 - 0.86)	0. 21

FeNa	1.89 (1.1 - 2.66)	0.64	(0.48-0.79)	0.75	(0.64-0.86)	0.21

Blood urea nitrogen (mmol/L)	32.5 (27.8 - 36.1)	0.66	(0.51-0.81)	0.77	(0.66 - 0.87)	0.18

UOsm (mosm/L)	382 (347 - 421)	0.74	(0.6 - 0.87)	0.71	(0.59 - 0.82)	0.1

UPCR	21.3 (17.8 - 29.5)	0.64	(0.49 - 0.78)	0.75	(0.65 - 0.86)	0.23

Urinary sodium (mmol/L)	51 (39 - 56)	0.61	(0.45 - 0.76)	0.65	(0.53 - 0.77)	0.02

UCR	28.6 (23.1 - 31.9)	0.56	(0.41 - 0.72)	0.60	(0.48 -0.72)	0.0001

FUN	36 (21 - 41)	0.37	(0.21 - 0.53)	0.41	(0.28 - 0.54)	<0.0001

Plasma bicarbonate (mmol/L)	13 (11.8 - 13.6)	0.64	(0.52 - 0.77)	0.70	(0.59 - 0.82)	0.09

Plasma potassium (mmol/L)	4.7 (4.4 - 5.1)	0.44	(0.26 - 0.61)	0.65	(0.54 - 0.77)	0.02

Urine NGAL (ng/ml)	644 (445 - 780)	0.62	(0.44 - 0.79)	0.68	(0.55 - 0.80)	0.045

* comparison of AUC in patients who should have had dialysis with plasma creatinine, using chi squared.

**Table 7:** Sensitivity, specificity, and the positive and negative predictive values of various selected laboratory tests performed on admission to predict the later requirement for dialysis^: optimal cut-offs in this series

Index	Optimal cut off**	Sensitivity	Specificity	PPV	NPV
Plasma creatinine μmol/L	202	77	78	75	80

RFI	1.4	71	72	69	74

FeNa	1.88	50	91	83	67

Urine Osmolality mosm/L	419	63	56	56	63

UPCR	43	84	44	57	76

Urinary Sodium mmol/L	34	90	44	59	83

Bicarbonate	15	82	56	62	78

NGAL	510	65	70	65	70

AUC: Area under receiver operating characteristic curve, RFI: Renal failure index, FeNa: Fractional excretion of sodium, UPCR: Urine: plasma creatinine ratio, FUN: Fractional excretion urea. NGAL: neutrophil gelatinase-associated lipocalcin.

** Determined using the ROC curve

^ Patients who should have had dialysis.

## Discussion

In this series of 163 patients the incidence of death and renal impairment was similar to a large multinational series of adults suffering from severe malaria [1]. It is likely then that it was a representative sample of adult patients suffering from MARF in a resource poor area of unstable malaria transmission.

In patients with renal impairment on admission, none of the evaluated parameters was superior to the simple measurement of plasma creatinine measurement in predicting a later requirement for RRT. Plasma creatinine has the additional advantage of being widely used and understood by clinicians and of being relatively inexpensive. It requires no mathematical derivation and has additional established uses including the adjustment of drug dosages. The other laboratory tests of ATN/AKI assessed in this study often require additional specimen collection, mathematical calculation and invariably entail additional costs. They do not appear to improve significantly the ability of clinicians to predict a later requirement for RRT. In busy, resource-poor settings they are thus tests of poor discriminatory value.

The series confirms that prompt RRT in the management of MARF reduces mortality. In the 19 (58%) patients who had an indication for RRT and who were able to receive this intervention, the mortality rate was 37%: significantly lower than the mortality rate in those who were unable to receive RRT (75%, p = 0.01) and no higher than the rate seen in patients in the series without ARF. This is despite the fact that only two of the patients were able to receive the more effective haemodialysis [4]. As in previous studies the duration of required peritoneal dialysis was relatively brief [3]. Furthermore, two of the patients who received RRT and died had a significant delay in starting dialysis: receiving it 24 hours after an indication was met.

Patients were unable to receive RRT for a range of reasons; all the consequence of limited resources: dialysis equipment was periodically unavailable (for financial or logistical reasons), intermittently there was a shortage of skilled staff to assess patients and administer and supervise dialysis and, occasionally, there was limited access to diagnostic facilities to assess the indications for dialysis. Such phenomena are not unusual in regions where patients with MARF will be managed.

Although almost half of the patients in the studies presented to hospital with a CrCl < 60 ml/min, only 46% of these would later require RRT, demonstrating that the majority of patients with malaria presenting with renal impairment will respond to anti-malarial chemotherapy and fluid repletion alone. As in previous studies, patients with renal impairment were morel likely to demonstrate evidence of hepatic dysfunction [3]. Only 22/43 patients who had an indication for dialysis had their urine output recorded, however, no more than 8 of these were oliguric. This reinforces the observations made by other investigators that MARF may be non-oliguric [3] and it underlines the unreliability of this clinical feature in identifying patients who may require RRT. This, together with the difficulty in obtaining reliable 24 hour collections and the obligatory delay, calls into question the utility of the WHO definition of malarial acute renal failure as "a plasma creatinine concentration >265 μmol/L with 24 h urine output of <400 ml" [2].

In a recent meta-analysis of critically ill patients the measurement of NGAL had an AURROC of 0.78 (95% CI 0.65-0.92) when predicting a requirement for RRT [15]. The performance of urinary NGAL in this series was similar (0.68 (95% CI 0.55-0.80) and was strongly correlated with plasma creatinine (p < 0.0001, r_s _= 0.64). However, while the optimal cut-off and timing of specimen collection in different patient groups with AKI has yet to be established [14,15,23], at this stage the knowledge of a urinary NGAL does not appear to significantly improve the ability of the clinician to predict a requirement for RRT in this population.

Plasma UCR appears to have a limited role in assessing the volume state of patients with severe malaria. Elevated BUN concentrations, reflecting the hypercatabolic state associated with severe malaria [24], lead to numerically greater UCRs. This finding may also have affected the performance of the FeUN test. We had anticipated that the elevated plasma urea would be corrected for by analysis of the fractional excretion of urea because in ATN impaired passive reabsorption of urea leads to an increase in the fractional excretion of urea. However in our series we found that the FeUN in patients who would later require dialysis was actually significantly *lower*. A suggested mechanism is that in hypercatabolic states the osmotic effect of higher tubular concentration of urea complicates the calculation of FeUN [7]. Indeed, more than 60% of the patients in the analysis had a urine urea:creatinine ratio greater than 10, the usual upper range of normal for this value.

There were several deficiencies in the study. A retrospective definition of whether a patient should have received RRT was the primary reference for analysis. Patients were classified from case files that were not prepared prospectively to collect these data which were sometimes incomplete. However the files were reviewed independently by three experienced clinicians and after discussion, there was unanimous consensus. The fact that over 90% of patients met criteria for RRT in the first 72 hours of their admission suggests that RRT therapy was required for MARF rather than a complication of inpatient management. Indeed when the four patients who met criteria for RRT after 72 hours of admission were removed from the analysis, the performance of all the laboratory indices was almost identical. A requirement for RRT was also no different in the patients enrolled in each of the two trials arguing against a confounding effect of NAC or levamisole. Although there are data supporting the efficacy of NAC in preventing radiocontrast nephropathy [25], there are no data suggesting a benefit of NAC in critical illness nephropathy [26,27]. Levamisole is not known to have any major effect on renal function [28]. Specimens were collected on admission before the administration of NAC or levamisole precluding the possible influence of either agent on any of the laboratory indices.

By classifying renal impairment as a plasma creatinine greater than 200 μmol/L in our criteria for dialysis, we biased our analysis in favour of plasma creatinine defining the dialysis population. However, plasma creatinine performed significantly better than BUN (another measure of renal function) and plasma bicarbonate - the criterion that actually qualified the patient for dialysis in 85% of the cases. Plasma creatinine is also used to define FeNa, FUN, RFI and UPCR, yet despite these indices measuring a second parameter of tubular function they performed no better than plasma creatinine alone. The plasma creatinine was not available to the clinicians managing the patients during their admissions and thus was not used to define the 19 patients who received RRT. If analysis is restricted to the patients who actually received RRT, plasma creatinine still has a greater AUROC than all other indices except urinary osmolality and the difference between these two variables is not significant. Although the figure of 200 μmol/L was very similar to the optimal cut-off for dialysis in this series of 202 μmol/L, the figure of 200 μmol/L was defined before the analysis. Acidosis and convulsions are also manifestations of severe malaria without renal impairment, hence using them as criteria to define the patients who "should have had RRT" is potentially flawed. However, as noted above, plasma creatinine was superior to plasma bicarbonate as a predictor of RRT, only one patient was dialysed because of convulsions, and this was in the setting of declining renal function.

There were no scales available for weighing obtunded patients thus bedside estimations of weights were used to derive the creatinine clearance (CrCl). Although these measures are clinically realistic, they introduce a potential error into the calculation. Furthermore, formulae used to estimate creatinine clearances are designed for use in patients with *chronic *renal impairment and our series composed of ethnic Bangladeshis in whom the Cockcroft and Gault formula has not been formally validated. However, we felt that it was best to use a mechanism that would control to some degree for the range of body types that were included in the series. Comparisons between the young muscular men and older women in the series may have been equally deficient if using the plasma creatinine as the reference for glomerular filtration rate.

For a new diagnostic test to be implemented it should be more sensitive than the existing diagnostic methods and it should not be more costly, less safe or less specific [29]. The measurement of plasma creatinine on admission in patients with severe malaria is safe and relatively inexpensive. In this series of patients it was able to predict the later requirement for RRT as accurately as the more complicated and expensive tests used to diagnose ATN/AKI. From these data there appears little to recommend the use of these tests in the resource poor settings where most patients with severe malaria will be managed.

## Conflict of interest

The authors declare that they have no competing interests

## Authors' contributions

JH conceived the study, managed the patients, performed the statistical analysis and was the primary author of the manuscript. MMUH, SA, EBY, MBF, NJW and NPD supervised the study. AAR performed laboratory analysis of specimens. PC managed the patients and assisted with the statistical analysis. RJM, STD managed the patients, MJS and MLM revised the manuscript. AMD was the senior supervisor assisted with the statistical analysis and revised the manuscript. All authors read and approved the final manuscript.
